# Opposing roles for amygdala and vmPFC in the return of appetitive conditioned responses in humans

**DOI:** 10.1038/s41398-019-0482-x

**Published:** 2019-05-21

**Authors:** Claudia Ebrahimi, Stefan P. Koch, Charlotte Pietrock, Thomas Fydrich, Andreas Heinz, Florian Schlagenhauf

**Affiliations:** 1 Department of Psychiatry and Psychotherapy, Charité – Universitätsmedizin Berlin, corporate member of Freie Universität Berlin, Humboldt-Universität zu Berlin, and Berlin Institute of Health, 10117 Berlin, Germany; 2 Department of Psychology, Humboldt University of Berlin, 10099 Berlin, Germany; 3 Cluster of Excellence NeuroCure, Charité – Universitätsmedizin Berlin, corporate member of Freie Universität Berlin, Humboldt-Universität zu Berlin, and Berlin Institute of Health, 10117 Berlin, Germany; 4 Max Planck Institute for Human Cognitive and Brain Sciences, 04303 Leipzig, Germany

**Keywords:** Learning and memory, Human behaviour

## Abstract

Learning accounts of addiction and obesity emphasize the persistent power of Pavlovian reward cues to trigger craving and increase relapse risk. While extinction can reduce conditioned responding, Pavlovian relapse phenomena—the return of conditioned responding following successful extinction—challenge the long-term success of extinction-based treatments. Translational laboratory models of Pavlovian relapse could therefore represent a valuable tool to investigate the mechanisms mediating relapse, although so far human research has mostly focused on return of fear phenomena. To this end we developed an appetitive conditioning paradigm with liquid food rewards in combination with a 3-day design to investigate the return of appetitive Pavlovian responses and the involved neural structures in healthy subjects. Pavlovian conditioning (day 1) was assessed in 62 participants, and a subsample (*n* = 33) further completed extinction (day 2) and a reinstatement test (day 3). Conditioned responding was assessed on explicit (pleasantness ratings) and implicit measures (reaction time, skin conductance, heart rate, startle response) and reinstatement effects were further evaluated using fMRI. We observed a return of conditioned responding during the reinstatement test, evident by enhanced skin conductance responses, accompanied by enhanced BOLD responses in the amygdala. On an individual level, psychophysiological reinstatement intensity was significantly anticorrelated with ventromedial prefrontal cortex (vmPFC) activation, and marginally anticorrelated with enhanced amygdala-vmPFC connectivity during late reinstatement. Our results extend evidence from return of fear phenomena to the appetitive domain, and highlight the role of the vmPFC and its functional connection with the amygdala in regulating appetitive Pavlovian relapse.

## Introduction

Learning about environmental cues that signal desirable outcomes constitutes an important mechanism to flexibly adapt behavior and foster survival. However, learning theories of addiction and obesity emphasize the persistent power of Pavlovian reward cues (conditioned stimuli, CS+)—a beer brand label in the super market or the smell of a freshly-baked cake—to trigger the desire for the associated drug/food (unconditioned stimulus, US), drive habits, and increase the risk of relapse long after abstinence^1–4^.

Although extinction—repeatedly presenting a CS+ without the US—reduces conditioned responding^5^, it does not “erase” the original cue-reward association, but induces new, highly context-dependent inhibitory learning^6^. Several Pavlovian relapse phenomena—a return of conditioned responding towards the extinguished CS+—originate from these properties of extinction, including the mere passage of time (spontaneous recovery), an unpredicted encounter with the US (reinstatement), or a change in context (renewal)^6,7^. Reinstatement, the return of conditioned responding towards an extinguished CS after an unpredicted encounter with the US, is well documented in rodents^8,9^.

From a clinical perspective, this challenges the efficacy of cue-exposure therapy to prevent relapse despite reducing cue reactivity in the clinic^10,11^. Translational laboratory models of human Pavlovian relapse could therefore represent a valuable tool to investigate the mechanisms that mediate relapse and develop new techniques to counteract it^12^. Anxiety research experimentally investigates return of fear following extinction on multiple response systems, including psychophysiological measures (skin conductance, fear-potentiated startle) and neuroimaging^13–15^. Conversely, experimental research on appetitive Pavlovian conditioning and relapse in humans is still in its infancy^16–18^. This research gap has been explained by difficulties to find universally-rewarding USs and a lack of established measures sensitive to appetitive responses^19–21^.

To this end we developed a differential delay conditioning paradigm with liquid food as natural unconditioned stimulus (US) in combination with a 3-day design to evaluate conditioning (day 1), extinction (day 2) and return of conditioned responding following a reinstatement procedure (3 day) while allowing consolidation of learning between sessions. A multimethod approach was used to assess conditioned responses, including explicit (CS pleasantness and US contingency ratings), behavioral (reaction times), and implicit measures (SCRs, heart rate, startle responses). Moreover, return of appetitive conditioned responses was investigated on a neuronal level using fMRI.

Preclinical work points toward an important role of the ventromedial prefrontal cortex (vmPFC) in mediating appetitive Pavlovian relapse after extinction^22,23^, suggesting it as a central site of extinction memory storage^24^. This regulatory role is accomplished via projections to key structures involved in reward-related learning, particularly basolateral amygdala and nucleus accumbens (NAcc)^25,26^. Corroborating animal findings, human neuroimaging confirmed the involvement of the amygdala as well as the ventral striatum, including the NAcc, during appetitive Pavlovian learning with primary rewards^27,28^. Furthermore, vmPFC activation was related to inhibiting previously learned appetitive responses^29^, possibly through enhanced functional connectivity with the amygdala^28,30^. In the fear domain, neuroimaging highlighted an important role for the vmPFC in extinction recall^31^, and amygdala, hippocampus and vmPFC have been shown to be differentially involved in reinstated fear^32,33^.

The aims of this study were twofold: (1) to test the hypothesis that appetitive conditioned responses in healthy subjects recover after a reinstatement procedure 24 h after extinction learning; (2) to characterize the neural structures involved in appetitive reinstatement and their relation to individual differences in reinstatement intensity. Based on the outlined findings, we hypothesized that amygdala, NAcc and vmPFC mediate reinstatement and that inhibition of conditioned responses depends on functional amygdala-vmPFC connectivity^30^.

## Materials and methods

### Subjects

Seventy-one healthy, right-handed volunteers recruited from student mailing lists gave written informed consent to participate in the appetitive conditioning session (day 1); the last 36 were followed up for extinction (day 2) and a reinstatement test (day 3). Participants were excluded in case of current or past medical, psychiatric or neurological disorders, drug or alcohol abuse, pregnancy, color blindness or weakness, being on a diet, and allergies or food intolerances to the delivered liquid foods (self-report). All subjects were right-handed and had normal or corrected-to-normal vision. Subjects were required to fast for at least four hours before each session^34^. A priori defined inclusion criteria (see ‘*Behavioral data acquisition*’) resulted in a final sample of 62 subjects during conditioning (*M* (SD)_age_ = 24.42(3.28) years; 35 women) and 33 subjects during extinction and reinstatement test (*M* (SD)_age_ = 24.06(3.81) years; 18 women). The study was approved by the Ethics Committee of the Charité – Universitätsmedizin Berlin and conducted in accordance with the Declaration of Helsinki.

### Experimental procedure

**Fig. 1:**
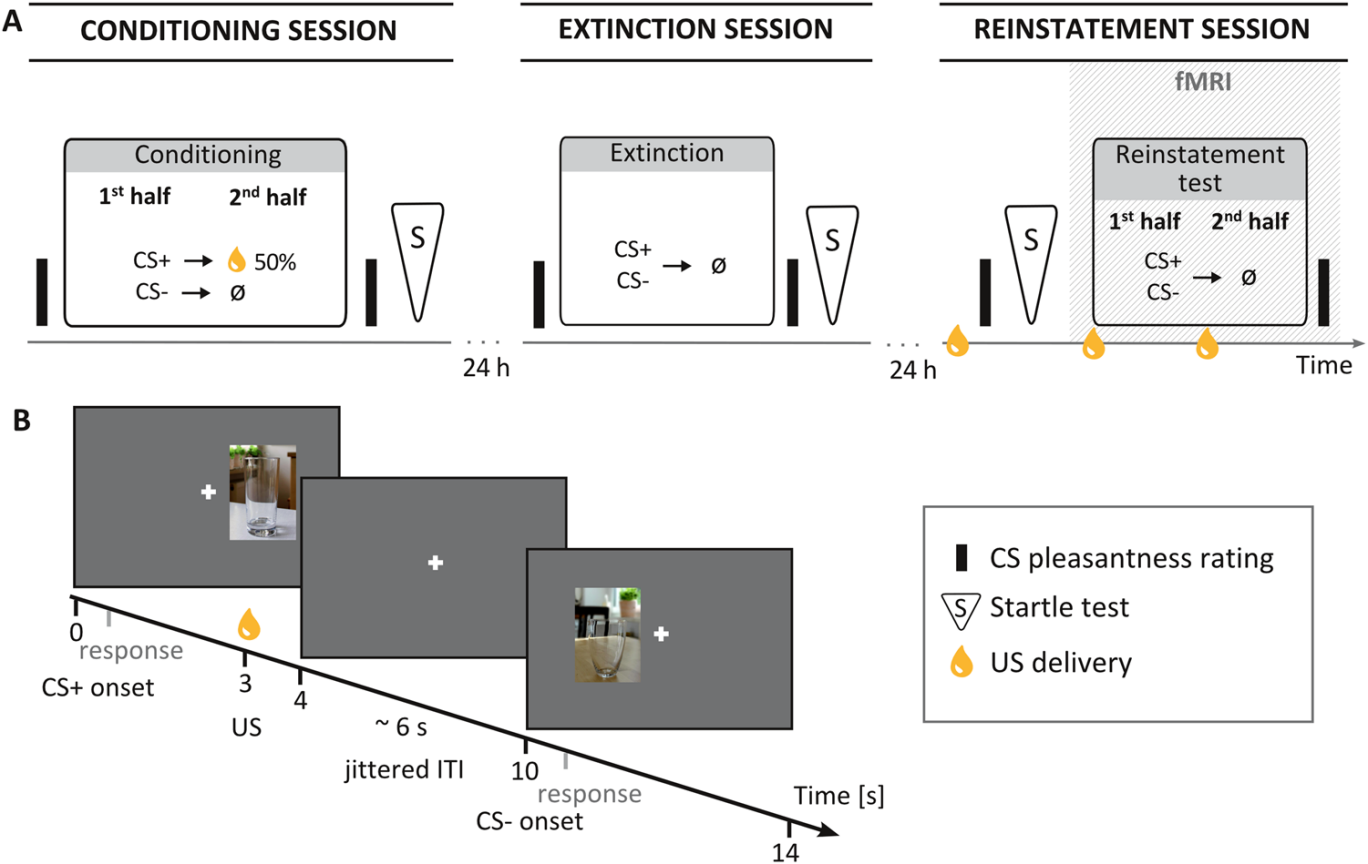
Experimental design and paradigm. **a** Subjects underwent appetitive differential delay conditioning on day 1, extinction on day 2, and a reinstatement test on day 3. Acoustic startle tests were conducted separately in each session. Return of conditioned appetitive responses on day 3 was probed after a reinstatement procedure occurring once before the startle test and twice during fMRI reinstatement test. **b** Exemplary trial sequence during conditioning on day 1. In each trial, one out of two different cues was presented either on the left or right side of a fixation cross for 4 s and subjects were asked to indicate the stimulus presentation side as fast as possible via button press. In case of a reinforced CS+ trial, 1 ml of subjects’ chosen liquid food (US) was delivered 3 s after cue onset

We used a differential delay conditioning paradigm with liquid food as natural unconditioned stimulus (US) with a multimethod approach over three sessions (conditioning, extinction, and reinstatement; Fig. 1a), 24 h apart to allow consolidation between sessions. Return of conditioned responding on day 3 was assessed after confronting subjects with unsignaled US presentations (reinstatement procedure). We provide publically available source code of the paradigm via Bitbucket (https://bitbucket.org/LearningAndCognition/appreinstatement).

#### Stimuli

One of four possible juices/smoothies (apple, orange, orange-passionfruit, strawberry-banana) served as US, depending on subject’s preference. US administration consisted of 1 ml of liquid delivered directly into the subjects’ mouth via clear PVC tubes and a programmable syringe pump (World Precision Instruments, Inc., Sarasota, USA). Two different pictures combined with one of two possible tones (400 or 500 Hz; 100-ms duration) served as cues (CS+/CS−, counterbalanced across subjects; see Fig. 1b).

#### Instructions

Subjects underwent uninstructed conditioning, extinction, and a reinstatement test, i.e., no information about CS-US contingencies or changes across sessions was provided. Until a final debriefing, subjects were told they were adult controls in an experiment investigating hand-eye coordination and attention in small children, where juice served to keep children’s motivation during the experiment. Subjects were asked to indicate the side of cue appearance via button press with their right index and middle fingers as quickly and accurately as possible.

#### Design

Conditioning (day 1) consisted of two phases, each with 30 CS+ and 30 CS− trials (total, 120 trials). In each trial, a cue was presented for 4 s to the left or right side of a white fixation cross. The CS+ was paired with US delivery 1 s before cue offset in 50% of the trials; the CS− was never followed by liquid food. To extinguish conditioned responses acquired on day 1, extinction (day 2) comprised one phase with 30 unreinforced CS+ and 30 CS− trials. The reinstatement test (day 3, in MRI) consisted of two identical phases, each with 15 unreinforced CS+/CS− trials. To trigger return of conditioned responding, subjects received three unsignaled, jittered US deliveries within 30 s prior to each phase (reinstatement procedure). Of note, due to the temporal spacing between sessions (24 h) and context changes from laboratory to scanner, spontaneous recovery and renewal might contribute to return of conditioned responding following reinstatement, providing a more ecologically valid model of appetitive Pavlovian relapse, where reinstatement effects inevitably follow some time after treatment (spontaneous recovery) and likely occur in a context different from initial acquisition (renewal). Trial sequences were pseudo-randomized across subjects (see Supplementary Material for further details). The inter-trial interval (ITI) ranged 3.5–12 s (*M* = 6 s).

### Behavioral data acquisition and preprocessing

#### Thirst and hunger ratings

Thirst and hunger ratings were collected prior to each session on separate 100-mm visual analog scales (VAS) ranging from 0 = ‘not thirsty/hungry at all’ to 100 = ‘very thirsty/hungry’.

#### Pleasantness of US and CS

US pleasantness was evaluated pre- and post-conditioning, and before the startle test on day 3 by applying a single US delivery, followed by a computerized VAS ranging from −50 = ‘very unpleasant’ to 50 = ‘very pleasant’. Only subjects with mean positive ratings pre-/post-conditioning were included in the study, ensuring US appetence during learning (subjects excluded: *n* = 6). On-screen pleasantness ratings for CS+ and CS− were acquired on an identical VAS before and after each session (Fig. 1a).

#### Contingency awareness

After conditioning, subjects rated the reward probability of each CS on a 100-point VAS (‘Immediately after this picture, I received a sip of juice…’) ranging from ‘never’ to ‘always’. Difference scores [CS+ *minus* CS−] were calculated as a dimensional awareness indicator, with large positive values implying contingency awareness and values around zero unawareness^35^. Subjects with notable negative difference scores (≤−20) were excluded from the study (*n* = 3), as these indicate (explicit) conditioning towards the CS− rather than unawareness. Following a worthwhile reviewer comment, we further explored associations between contingency awareness and conditioning indices on day 1 (see Supplementary Material).

### Psychophysiological data acquisition and preprocessing

Psychophysiological data were acquired at 250 Hz using an MR-compatible amplifier (BrainAmp ExG, Brain Products, Munich, Germany).

#### Skin conductance

Skin conductance responses (SCRs) were recorded during all sessions from the participant’s middle phalanges of the left index and middle finger using MR-compatible Ag/AgCl electrodes. Two datasets were excluded because of technical failures during recording or low data quality. Preprocessing and analysis of single-subject data was performed within the PsPM toolbox (version 3.1.1; http://pspm.sourceforge.net/), using the general linear convolution model (GLM) approach. Preprocessing comprised linear interpolation of movement-related artefacts, band-pass filtering (first-order Butterworth, 0.05–5 Hz), downsampling (10 Hz), and normalization to remove between-subject variance in response amplitudes^36^. Event onsets (CS+/CS−/US) were modeled as stick functions and convolved with a canonical SCR function. GLMs for conditioning and extinction included CS+ and CS− onsets per phase as regressors of interest, and an additional regressor to model the US effect on day 1. To test for return of conditioned responses on day 3, cue onsets of the first five CS+ and CS− trials following unsignaled US deliveries in each phase were modeled as two regressors of interest. US onsets and the remaining cues were modeled as three regressors of no interest. Within each GLM, regressors were fitted to the SC time series, yielding an SCR-amplitude estimate per regressor.

#### Startle responses

Cue-related modulation of eyeblink and postauricular (PAR) reflexes was assessed by auditory startle tests in each session (Fig. 1a). During the reinstatement session, three unsignaled US deliveries (reinstatement procedure) preceded the startle test. Startle probes consisted of a 50-ms, 90-dB burst of white noise presented binaurally via headphones. The test was initiated by four jittered startle probes while viewing the fixation cross (habituation startles; mean inter-probe-interval 2 s). Thereafter, startle probes were presented in each of four unreinforced CS+/CS− trials with varying stimulus-onset asynchronies (0.5, 1, 1.5, or 2 s). Four additional startle probes were presented during the ITI to reduce startle predictability and avoid a cue-related association. The ITI ranged 9–13 s (*M* = 10.9 s). Trial order and stimulus side were fully counterbalanced across subjects by assigning one of four fixed trial sequences. Startle responses were recorded with four Ag/AgCl electrodes on left orbicularis oculi and auricularis muscles^37,38^. Due to technical reasons, startle data from the first 12 participants are missing, and two datasets from the conditioning sample were lost because of technical failures. Electromyography (EMG) signal was notch-filtered at 50 Hz and band-pass filtered (2nd order Butterworth, 28–110 Hz), after which data were rectified and smoothed (3rd order Savitzky-Golay filter) using a moving average of 15 and 20 consecutive data points for PAR and eyeblink reflex, respectively. Startle responses were defined as the difference between the maximum amplitude within 20–115 ms and 10–40 ms after probe onset for eyeblink reflex and PAR, respectively, and startle baseline, i.e., mean EMG activity 50 ms before probe onset (ranges comparable to^37,38^), and averaged over cue type for group analyses. Eyeblink data from two subjects on day 1 and from one subject on day 3 had to be excluded for low quality.

Acquisition and preprocessing of heart rate (HR) and reaction times (RTs) is provided in the Supplementary Material.

### Imaging data acquisition and preprocessing

On day 3, MR data were acquired on a 3 Tesla scanner (Trio, Siemens AG, Erlangen, Germany) using a 32-channel head coil with a standard EPI sequence (40 slices, 3 × 3 mm^2^ in-plane voxel resolution, TR = 2.09 s, TE = 22 ms, 90° flip angle, 64 × 64 matrix; 192 × 192 mm FOV). Preprocessing and statistical analyses were performed within SPM12 (www.fil.ion.ucl.ac.uk/spm/) implemented in Matlab R2015b (The MathWorks, Inc., Natick, Massachusetts, United States). Preprocessing included slice time correction, realignment to the mean EPI volume, unwarping using the acquired field map, segmentation of the structural T1 image, coregistration of the segmented structural image to the mean EPI, spatial normalization to MNI space based on normalization parameters derived from each subjects’ structural image (2-mm isotropic voxel resolution), and smoothing using a 6-mm full-width at half maximum Gaussian Kernel.

### Statistical analysis of behavioral and physiological parameters

#### Effects of conditioning and extinction

CS pleasantness ratings during conditioning and extinction were analyzed separately with repeated measures ANOVA (rmANOVA) with within-subject factors cue (CS+/CS−) and time (pre/post). SCR, HR and RT during conditioning were analyzed analogously, with the factor time referring to early/late phase of conditioning. Paired *t*-tests were used to analyze cue differences during the extinction phase and all startle tests.

#### Reinstatement effects

Differential responding on day 3 was only probed for measures showing a significant conditioning effect on day 1 and successful extinction on day 2. Differential valence ratings and startle responses were evaluated using paired *t*-tests. For the fMRI reinstatement test, SCRs were estimated for the first five trials per cue after each reinstatement (10 trials/cue) and analyzed using a paired *t*-test.

Statistical analyses were performed within R version 3.4.3^39^ and rmANOVAs were Greenhouse-Geisser-corrected when necessary. Significant interaction effects were followed by *post-hoc* *t*-tests. The alpha level was set at .05 for all analyses and effect sizes were estimated using partial Eta^2^ (*η*^2^*p*) and Cohen’s *d*.

### Statistical analysis of imaging data (day 3)

An event-related analysis was applied using SPMs GLM approach on two levels. On the subject level, onsets for CS+ and CS− were included for each phase after convolution with the canonical HRF. US onsets and movement parameters were entered as regressors of no interest. Baseline contrasts for CS+ and CS− were computed for each phase and entered into a flexible factorial model on the group level. Reinstatements effects were analyzed by contrasting CS+ vs. CS− across phases. Possible time effects during the reinstatement test were investigated by assessing the cue × phase interaction. Based on evidence showing amygdala and NAcc involvement in appetitive Pavlovian conditioning^27,28,40^ and the role of the vmPFC in successful extinction recall^41,42^ and reinstated fear^32,33^, we applied small volume correction for amygdala, NAcc, and vmPFC at *p* ≤ .05 FWE-corrected. In our sample (*n* = 33) we had a power of 0.88 to detect medium effects (*d* = 0.5) at this threshold (G*Power 3^43^). Bilateral amygdala and NAcc masks were derived from WFU PickAtlas (http://www.fmri.wfubmc.edu/download.htm). For the vmPFC mask, a 10-mm sphere centered on [x = 0, y = 40, *z* = −12] was used based on previous studies on reinstated fear^32,33^.

For completeness, exploratory whole-brain analyses at *p* < .001 uncorrected are provided in the Supplementary Material.

*gPPI analysis*. Based on previous findings^30^, we investigated the interplay between amygdala and vmPFC during the reinstatement test and analyzed their cue-dependent functional connectivity using generalized psychophysiological interaction (gPPI) analysis (gPPI toolbox, http://www.nitrc.org/projects/gppi)^44^. For each participant, the first eigenvariate time series was extracted from the left amygdala seed and deconvolved to generate the neuronal signal^45^. For each cue type and phase, a PPI term was created by multiplying the respective cue onsets with the neural time series and convolving it with the HRF. The four PPI terms and the seed region time course then entered as regressors into first-level models otherwise similar to the primary analysis first-level GLM. Estimated connectivity parameters for each cue type and phase were analyzed in SPM’s full factorial design on the second level. Cue-specific connectivity was analyzed by contrasting PPI terms for CS+ vs. CS- across phases, and time-dependent changes by interacting cue differences with phase. A region-of-interest (ROI) analysis for the vmPFC was applied at *p* ≤ .05 FWE-corrected, following the proposed modulatory influence from vmPFC on amygdala activity during aversive^41,42^ and appetitive^30^ extinction recall.

*Associations with psychophysiological reinstatement effects*. In order to link the observed psychophysiological reinstatement effect (SCRs) to brain activation, simple regression analyses were performed within SPM introducing differential SCRs during reinstatement test as a covariate using the contrast images ‘CS+ vs. CS−’, as well as cue-specific connectivity differences (CS+ vs. CS−) from the gPPI analysis in separate SPMs. These analyses were complemented by subgroup analyses based on a median split of differential SCRs, thereby contrasting a low reinstatement (*n* = 17) with a high reinstatement group (*n* = 16).

Following a reviewer’s suggestion, an exploratory gPPI analysis using the right NAcc as seed region was applied (see Supplementary Material).

## Results

### Manipulation checks

#### Thirst and hunger

Ratings confirmed thirst and moderate hunger before conditioning (thirst: *M* (SD) = 63.7(20.6); hunger: *M* (SD) = 43.3(27.1)), extinction (thirst: *M* (SD) = 68.7(21.8); hunger: *M* (SD) = 51.8(29.0)), and reinstatement test (thirst: *M* (SD) = 65.0(18.6); hunger: *M* (SD) = 48.4(26.6)).

#### US pleasantness

**Fig. 2:**
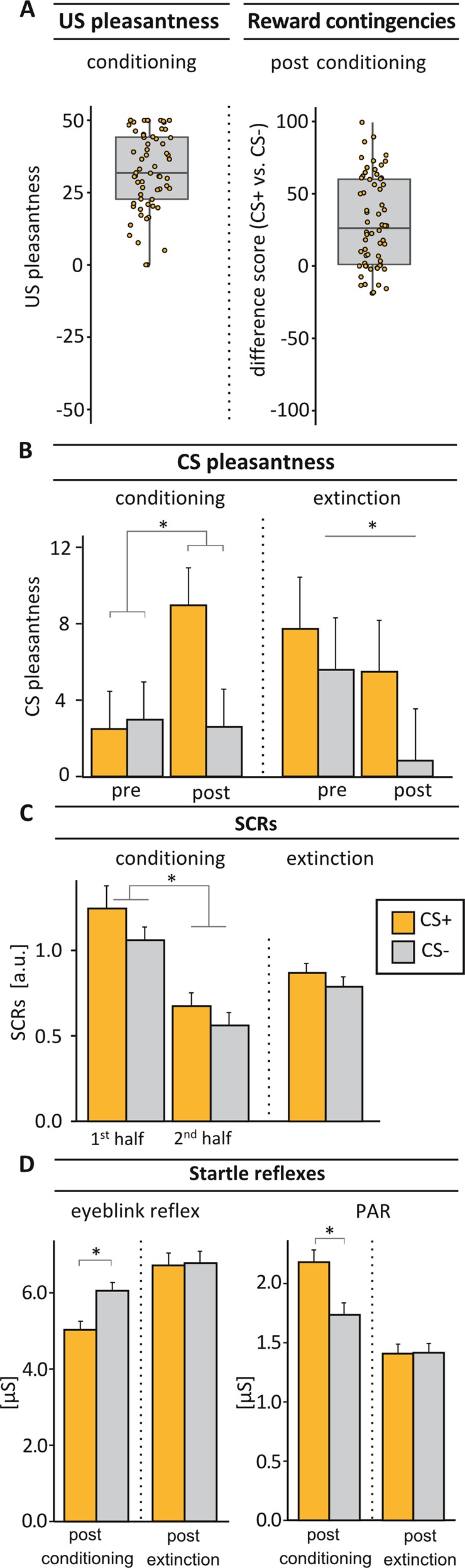
Indices of conditioning and extinction. **a** Study inclusion criteria of mean US pleasantness ratings (US pleasantness rating ≥ 0; left panel) and difference scores of rated reward contingencies (CS+*minus* CS− <−20; right panel) on day 1. **b** CS pleasantness ratings increased selectively for CS+ from pre to post conditioning, resulting in a significant cue × time interaction (*F* (1,61) = 4.32, *p* = .042). During extinction, a general decline in CS pleasantness was observed (main effect of time: *F* (1,32) = 4.93, *p* = .034). **c** Larger SCRs towards the CS + compared to the CS− across both acquisition phases were observed during conditioning (main effect of cue: *F* (1,59) = 7.08, *p* = .010). This differentiation was successfully extinguished on day 2 (*t*(32) = 0.99, *p* = .329). **d** Conditioning resulted in marked differences between startle responses during CS+ compared to CS− presentations in a subsequent acoustic startle test. While the eyeblink reflex was attenuated, the PAR was enhanced (*p* ≤ .005). Differential modulation of startle responses disappeared completely after extinction (*p* ≥ .894). Note that only a subsample of subjects participating on day 1 (conditioning sample) was further investigated during extinction and reinstatement test. For sample sizes in each measure, please see methods section. Error bars represent within-subject SEM^81,82^; a.u., arbitrary units; *p ≤ .05

The perceived pleasantness of the chosen juice/smoothie was high throughout conditioning (*M* (SD) = 32.32(13.60); Fig. 2a) and before the reinstatement test (*M* (SD) = 32.61(15.68)), and remained unchanged over sessions (day 1 vs. day 3: *Z* = −0.08, *p* = .935; Wilcoxon signed-rank test).

#### Contingency awareness

Reward contingencies for CS+ were rated significantly higher than for CS− after conditioning, indicating overall contingency knowledge across subjects that varied to different degrees (*M* (SD)_diff_ = 30.40(31.58), *t*(61) = 7.58, *p* < .001, range: −18.84 to 99.34; Fig. 2a, see Supplementary Material for further details), suggesting that uninstructed conditioning in addition to our cover story allowed for variability regarding explicit learning.

### Conditioning and extinction

#### CS pleasantness

Analysis of CS pleasantness during conditioning revealed a significant cue × time interaction (*F* (1,61) = 4.32, *p* = .042, *η*^2^*p* = 0.07; Fig. 2b). Closer inspection showed that subjective pleasantness of the CS+ increased significantly from pre- to post-conditioning (*t*(61) = −2.96, *p* = .004), while CS− pleasantness remained unchanged (*t*(61) = 0.13, *p* = .900), resulting in a trendwise differentiation between CS+ and CS− after acquisition (*t*(61) = 1.98, *p* = .052) but not at baseline (*t*(61) = −0.21, *p* = .840). During extinction, a significant main effect of time (*F* (1,32) = 4.93, *p* = .034, *η*^2^*p* = .13) indicated an overall decrease in CS pleasantness, but no cue × time interaction or main effect of cue (all *p* ≥ .413).

#### Skin conductance responses (SCRs)

During conditioning, SCRs towards the CS+ were significantly larger compared to the CS− across phases (main effect cue: *F* (1,59) = 7.08, *p* = .010, *η*^2^*p* = .11; Fig. 2c). We also observed a significant time effect (*F* (1,59) = 22.16, *p* < .001, *η*^2^*p* = .27) due to declining SCRs towards both cue types, but no cue × time interaction (*F*(1,59) = 0.53, *p* = .468). As expected, differential SCRs were no longer observed during extinction on day 2 (*t*(32) = 0.99, *p* = .329).

#### Startle reflexes

Successful conditioning was confirmed by a significantly attenuated eyeblink reflex (*t*(45) = −3.19, *p* = .003, *d* = −.47) as well as a significantly enhanced PAR (*t*(47) = 2.98, *p* = .005, *d* = .46; Fig. 2d) when contrasting startle reflexes during CS+ compared to CS− presentations post-conditioning. After extinction, neither eyeblink reflex nor PAR were differentially modulated by cue type, indicating complete extinction (all *p* ≥ .894).

#### Heart rate (HR)

Analysis of HR during conditioning revealed HR increases towards both cue types (main effect time: *F* (1,58) = 8.03, *p* = .006, *η*^2^*p* = .12) but no main effect of cue or cue × time interaction (*F* (1,58) ≤ 1.95, *p* ≥ .168). HR responses did not differ during extinction (*t*(32) = −0.82, *p* = .418).

#### Reaction times (RTs)

RTs obtained from the stimulus side detection task revealed no significant main or interaction effects during conditioning (*F* (1,60) ≤ 1.80, *p* ≥ .185) nor extinction (*t*(32) = −0.31, *p* = .760).

The reported conditioning effects remained unchanged when controlling for possible sample effects (conditioning sample vs. 3-day sample).

### Behavioral and psychophysiological reinstatement effects

**Fig. 3:**
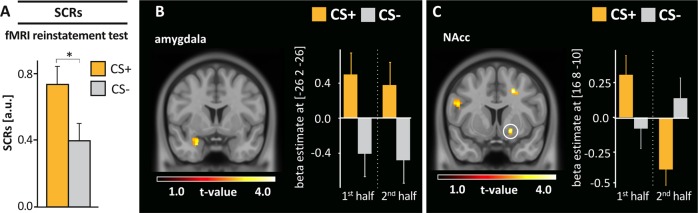
Psychophysiological and neural responses during reinstatement test. **a** Return of appetitive conditioned responses was observed during fMRI reinstatement test with significant larger SCRs towards the CS+ compared to the CS− during the first 5 CS+ and CS− presentations in both phases (*t*(32) = 2.25, *p* = .031). Error bars represent within-subject SEM^81,82^. **b** Elevated BOLD response in the contrast CS+ > CS− in the left amygdala over phases (MNI peak at [x: −26, y: 2, z: −26], *p*_FWE ROI_ = .01). **c** Interaction of differential BOLD responses with test phase in the right NAcc due to CS+ related activation declines from early to late reinstatement test (MNI peak at [x: 16, y: 8, z: −10], *p*_FWE ROI_ = 016). All t-maps are displayed on a visualization threshold of *p* < .005 uc with *k* ≥ 20 cluster extend

Return of appetitive conditioned responding was investigated for measures showing successful conditioning and extinction, i.e., CS pleasantness ratings, startle responses, and SCRs. Extinguished differences of eyeblink reflex or PAR did not recover at test (*p* ≥ .500). In contrast, following reinstatement in the fMRI, a significant return of conditioned responding was observed in SCRs, with larger SCRs towards CS+ compared to CS− (*t*(32) = 2.25, *p* = .031, *d* = .39; Fig. 3a). CS pleasantness ratings did not differ significantly at either time point (*p* ≥ .170), although CS+ pleasantness significantly increased from laboratory to fMRI reinstatement test (*t*(32) = −2.88, *p* = .007) while CS− pleasantness remained unchanged (*t*(32) = −0.83, *p* = .414).

### Neural responses during fMRI reinstatement test

The fMRI reinstatement test was accompanied by a significant differential BOLD response in the left amygdala with stronger activation towards the CS+ compared to the CS− ([x:−26, y:2, z:−26]; *Z* = 3.82; *p*_FWE ROI_ = .010, Fig. 3b). The inverse contrast (CS−>CS+) revealed no significant activation differences. We also looked for time dependent effects, as return of conditioned responding may decline with repeated unreinforced CS presentations despite the second reinstatement between phases. We observed a significant decline in differential BOLD response over time (cue × time interaction) in the right NAcc ([x:16, y:8, z:−10]; *Z* = 3.35, *p*_FWE ROI_ = .016; Fig. 3c) and trendwise also in the left amygdala ([x:−20, y:−6, z:−18]; *Z* = 2.23; p_FWE ROI_ = .064), but no differential BOLD response increase over phases.

### Neural responses associated with psychophysiological reinstatement effects

**Fig. 4:**
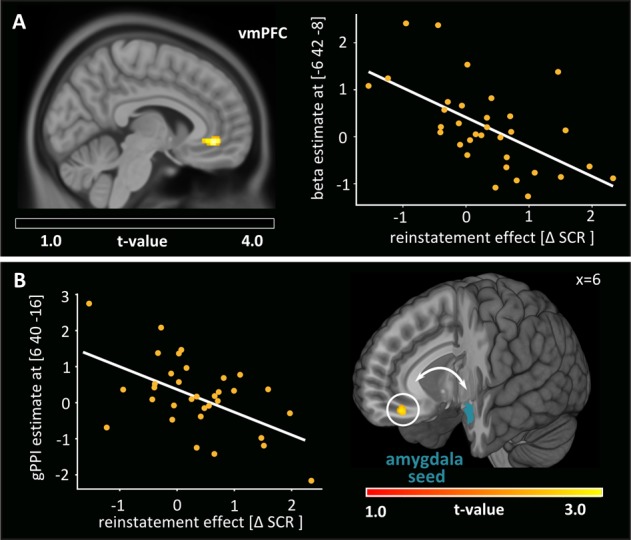
Neural responses associated with psychophysiological reinstatement effects. **a** Differential BOLD responses in the vmPFC during reinstatement test were inversely correlated with Pavlovian reinstatement intensity, indexed by differential SCRs (MNI peak at [x: −6, y: 42, z: −8], *p*_FWE ROI_ = .022). **b** Differential SCRs were further marginally inversely correlated with functional amygdala-vmPFC connectivity (gPPI) observed in the second test phase (MNI peak at [x: 6, y: 40, z: −16], *p*_FWE ROI_ = .061). Overlayed in cyan is the unilateral anatomical ROI mask of the left amygdala used to extract the time course of the seed region for the gPPI analysis. All t-maps are displayed on a visualization threshold of *p* < .005 uc with *k* ≥ 20 cluster extend

We investigated brain regions associated with the psychophysiological reinstatement effect by performing regression and subgroup analyses based on differential SCRs during the reinstatement test. Regression analysis revealed a significant negative correlation between differential SCRs and activation within the vmPFC ([x:−6, y:42, z:−8]; *Z* = 3.63; *p*_FWE ROI_ = .022; Fig. 4a), indicating stronger vmPFC activity towards CS+ compared to CS− in subjects showing attenuated differential SCRs. Corroborating this association, directly contrasting participants with high vs. low differential SCRs confirmed significant higher vmPFC activation in the low reinstatement compared to the high reinstatement group ([x:−4, y:42, z:−8], *Z* = 3.83, *p*_FWE ROI_ = .011; Fig. S1A).

#### Functional connectivity between amygdala and vmPFC

Based on the proposed inhibitory role of the vmPFC over amygdala to support successful extinction recall^30,41,42^, we further investigated cue-dependent functional connectivity between the amygdala and the vmPFC during the reinstatement test. The gPPI analysis showed that, while no significant amygdala-vmPFC connectivity was evident across phases, connectivity during CS+ compared to CS− presentation was significantly enhanced in the second phase of the reinstatement test ([x:8, y:44, z:−16]; *Z* = 3.49; *p*_FWE ROI_ = .032). Interestingly, this connectivity was further marginally anticorrelated with the psychophysiological reinstatement effect ([x:6, y:40, z:−16]; *Z* = 3.25; *p*_FWE ROI_ = .061; Fig. 4b), i.e., tended to be enhanced in participants with low compared to high differential SCRs during reinstatement test ([x:6, y:40, z:−16], *Z* = 3.08, *p*_FWE ROI_ = .077; Fig. S1B).

## Discussion

This study investigated the return of experimentally conditioned appetitive responses in healthy subjects as a translational laboratory model of appetitive Pavlovian relapse. We showed that SCRs recover after a reinstatement procedure 24 h after extinction and provide evidence for opposing roles of amygdala and vmPFC in mediating Pavlovian relapse. During the reinstatement test, amygdala activation towards the CS+ was enhanced, while psychophysiological reinstatement intensity was significantly anticorrelated with vmPFC activation and marginally with enhanced amygdala-vmPFC connectivity observed during late reinstatement.

### Amygdala and NAcc activity during appetitive Pavlovian relapse

The reinstatement test showed increased BOLD responses in the left amygdala towards CS+ compared to CS− presentations. Amygdala activity was present over both test phases, declining trendwise over time. Preclinical studies have demonstrated the central role of the amygdala in appetitive Pavlovian learning^25,46^ and cue-induced relapse in animal models of drug addiction^47^. Corroborating animal findings showing the relevance of the amygdala in the formation of CS-US associations^48^, human neuroimaging studies have repeatedly observed amygdala activation during appetitive Pavlovian learning with primary rewards^27,28,40^. The increased amygdala activation present in our study therefore likely reflects retrieval of the original CS−US association. In line with our results, enhanced amygdala activation towards a previously extinguished fear cue has also been observed following unsignaled aversive US presentations^32,33,49^ or context changes^50,51^. Enhanced amygdala activation has further been observed towards an extinguished monetary CS+ following a reactivation procedure 24 h after extinction in healthy controls^30^. We further observed time-dependent differential NAcc activity due to CS+ related declines in BOLD responses from early to late reinstatement, suggesting a more transient involvement of this structure during the reinstatement test. Animal and human work have identified the NAcc within the ventral striatum as a key structure in the brain’s reward circuit, being involved in reward processing and reward-related learning^52,53^. In rodents, expression of conditioned approach behavior towards food or drug cues depend on an intact NAcc^54–56^ and human neuroimaging has shown increased BOLD responses in the ventral striatum towards cues predicting primary rewards^27,28,40,57^.

### vmPFC mediated inhibition of appetitive conditioned responses

In contrast to enhanced amygdala and NAcc activity, we did not observe significant vmPFC activation towards CS+ compared to CS− presentations during the reinstatement test. Differential BOLD responses in this region were instead inversely related to psychophysiological reinstatement intensity (i.e., differential SCRs), whereby increased vmPFC involvement was only present in subjects experiencing weak and not in those showing strong reinstatement effects. This finding directly adds to animal evidence supporting a role for the vmPFC in inhibiting maladaptive learned associations^25,26^. Rodent studies have demonstrated that lesions of the infralimbic (IL) cortex as the homolog region of the human vmPFC do not impair acquisition or within-session extinction of appetitive Pavlovian responses, but impair retrieval of extinction the following day, resulting in increased spontaneous recovery, reinstatement, and renewal^22,23^. Conversely, optogenetic activation of IL neurons has been shown to suppress the return of appetitive conditioned responses^58^. Animal models of cue-induced reward seeking after extinction further revealed IL involvement in both drug and natural reward seeking responses^26,59,60^ and specific CS-responsive neuronal ensembles within the IL have been shown to exert inhibitory control over alcohol seeking^61^.

In line with our result, human neuroimaging has implicated the vmPFC in successful aversive extinction learning and recall^31–33,42,50,62^, and vmPFC activity^41,42,62^ and cortical thickness^63^ scaled inversely with conditioned SCRs. Our results further extend recent evidence linking reduced vmPFC involvement to return of fear following reinstatement^32,64^. In these studies, differential vmPFC activity was present during simple extinction recall but not after a reinstatement procedure^32^, and reduced CS− related vmPFC activity during reinstatement test compared to extinction recall was associated with increased SCRs, indicating a “release from inhibition”^64^. Our results further suggest an important regulatory role for the vmPFC in appetitive Pavlovian relapse. Adding to this, increased ventral vmPFC activity has been shown towards a cue no longer paired with a monetary reward, consistent with an inhibitory signal^29^.

The rodent vmPFC is widely connected^65^. Given its strong projections to the amygdala^66,67^ and previous findings on functional amygdala-vmPFC connectivity during appetitive extinction recall^30^, we investigated cue-dependent functional connectivity between amygdala and vmPFC during the reinstatement test. While there was no evidence of enhanced connectivity across phases, amygdala-vmPFC coupling during CS+ relative to CS− presentations was significantly enhanced during late reinstatement. On an individual level, amygdala-vmPFC connectivity might be further inversely related to the psychophysiological reinstatement effect, indicated by a marginally significant anticorrelation. These findings add to imaging studies reporting functional amygdala-vmPFC connectivity during fear extinction recall^41,42^ (but see^50^) and indicate that amygdala-vmPFC coupling constitutes an important neural correlate of successful extinction recall despite adverse circumstances. In line with this, enhanced cue-dependent amygdala-vmPFC coupling has been observed during appetitive extinction recall in subjects receiving the NMDA receptor agonist D-cycloserine hypothesized to enhance extinction consolidation compared to placebo^30^. Moreover, increased amygdala-vmPFC connectivity during initial appetitive conditioning seems to attenuate amygdala activity and acquisition of SCRs^28^. Our finding that amygdala-vmPFC connectivity only emerged during the late reinstatement test is consistent with the proposed disinhibition due to decreased vmPFC activity observed in reinstated fear^64^. Amygdala-vmPFC connectivity might therefore increase as reinstatement effects decline, in line with declining differential BOLD responses observed over test phases for NAcc and trendwise for amygdala.

### Psychophysiological and behavioral measures of conditioned responding

We observed a differential return of conditioned responding during the reinstatement test in an implicit measure (SCRs), providing evidence that human appetitive Pavlovian relapse can be modeled in the laboratory. Most of what is known about Pavlovian relapse effects in humans stems from investigations on return of fear phenomena^13,15,68^. The relative lack of translational research in the appetitive domain has been explained by difficulties in finding suitable USs and measures sensitive to appetitive conditioned responses^19–21^. In line with a previous study using food US^16^, we observed successful conditioning and extinction in pleasantness ratings, SCRs and eyelid startle, clearly indicating the validity of our design. Conditioning also resulted in a significantly enhanced PAR, thereby replicating recent evidence demonstrating the sensitivity of this microreflex as an appetitive conditioning index^20^. By contrast, HR and RTs did not provide sensitive indices of conditioning in our paradigm. Unlike SCRs, neither pleasantness ratings nor startle responses showed significant reinstatement effects. Diverging findings across multiple response measures are also commonly reported in fear reinstatement studies^13,69^, and multimodal investigation of appetitive conditioning suggests that different conditioning indices are only weakly related on an individual level^21^. We observed a rather weak conditioning effect in pleasantness ratings with only trendwise differentiation after conditioning. Explicit ratings might primarily reflect cognitive learning components^70^ as indicated by the influence of contingency awareness in our study (see Supplementary Material), while our uninstructed learning paradigm and cover story was intended to reduce cognitive demands^71^. Moreover, pleasantness ratings have been shown to be rather insensitive to extinction^70,72,73^ and, in contrast to SCRs or BOLD responses, might not distinguish experimental groups well^28,57^. While we observed a robust startle modulation after conditioning, participants may have learned to distinguish the non-reinforced and aversive startle context from appetitive acquisition in this session, potentially impeding assessment of startle modulation on subsequent days. Apart from that, the scanner environment possibly enhanced the return of conditioned responses observed during the fMRI reinstatement test, i.e., a stronger renewal effect might have added to the return of conditioned responding.

## Conclusions

Our finding that appetitive conditioned responses returned after unsignaled US presentations 24 h after extinction extends existing evidence on return of fear phenomena in humans to the appetitive research domain. Moreover, our results suggest opposing roles for amygdala and vmPFC in mediating appetitive Pavlovian relapse effects. This is of particular clinical relevance, as drug addiction is associated with heightened amygdala responses towards disease-related cues^74^, while, at the same time, addicted patients exhibit grey matter volume decreases and activation impairments in vmPFC^75–79^. Future studies could investigate appetitive Pavlovian conditioning and relapse phenomena in patients with addiction and explore inter-individual differences in these processes as well as its interaction with instrumental responding (e.g., Pavlovian-instrumental transfer (PIT)^80^). Although correlational, our findings suggest that the vmPFC could be a promising target for novel intervention techniques that aim to counteract appetitive Pavlovian relapse.

## Supplementary information

Supplementary Material
