# Supplementary material for: Opposing roles for amygdala and vmPFC in the return of appetitive conditioned responses in humans
Source: Transl Psychiatry. 2019 May 21;9:148. doi: 10.1038/s41398-019-0482-x (PMC6529434; doi:10.1038/s41398-019-0482-x)
Supplement: Supplementary file 1 — Supplementary Material [file 41398_2019_482_MOESM1_ESM.pdf]

Supplementary Material to:

Opposing roles for amygdala and vmPFC in the return of appetitive conditioned responses in humans

Claudia Ebrahimi, Stefan P. Koch, Charlotte Pietrock, Thomas Fydrich, Andreas Heinz, Florian Schlagenhauf

Paradigm and trial structure

Trial sequences were pseudo-randomized across subjects and sessions, but restricting the occurrence of identical cue type or stimulus side to a maximum of three successive trials. *Additional restriction conditioning*: Within subjects, both phases followed the same trial sequence except that the very first four trials were fixed to initiate learning equivalently across subjects (reinforced CS+, CS-, reinforced CS+, CS-).

Additional restriction reinstatement test: As reinstatement effects are assumed to be transient, each phase started with two alternating CS+/CS- presentations (either [CS+ CS- CS+ CS-] or [CS- CS+ CS- CS+]), counterbalanced across subjects.

The paradigm was programmed in Matlab (R2011a; The Mathworks, Natick, United States) using Cogent (Wellcome Department of Imaging Neuroscience, Institute of Neurology, London, UK) and presented on a 19” computer screen except during fMRI, when stimuli were presented on an MR-compatible LCD screen (32″, NNL LCD Monitor®, NordicNeuroLab, Bergen, Norway).

Data Acquisition and Preprocessing

Heart Rate. Heart rate (HR) during conditioning and extinction was measured using electrocardiography (ECG) with bipolar leads. Adhesive electrodes were placed in right parasternal costoclavicular space and left mid-clavicular line in the fifth intercostal space. The ground electrode was placed on the costal margin in the right mid-clavicular line. During the reinstatement test, photoplethysmography (PPG) attached to the left index finger was used as a measure of cardiac activity, recorded at 50 Hz via the Siemens Physiological Monitoring Unit. The QRS detection algorithm proposed by Pan & Tompkins[1] was used to extract QRS complexes from ECG data. For the PPG, online pulse period (PP) detection within the PMU was used. Subsequently, the complete time sequence with detected RR

intervals (PP intervals on day 3, respectively) was visually inspected and manually corrected, if necessary. Sequences with artefacts or low signal in the data preventing reliable heart beat detection were treated as missing data points. The time series of non-uniform inter-beat

intervals was converted to HR and interpolated to the sample rate of acquisition. Trials with missing data in a window from -1 to 4 s with respect to CS onset were excluded from further analyses. Mean HR was calculated for the time window 1-3 s after cue onset. Trialwise HR data were normalized and aggregated over each phase and cue type. In the conditioning sample technical failures during recording caused data loss in three subjects, while for day 3 another five subjects were excluded due to failed PP detection (low signal-to-noise ratio/ PPG dislocation). Because swallowing causes prolonged HR changes after US delivery[2], only trials without preceding reinforcement were considered for analysis of HR during conditioning (day 1).

Reaction Time. RTs from the cue side detection task were collected in the laboratory and fMRI with a 2-button keypad and MR-compatible response buttons, respectively. RTs between 200-2400 ms were considered valid responses. Data were log transformed to reduce skewness, and averaged over each phase and cue type. Data from one subject are missing due to technical malfunction.

Neural responses during fMRI reinstatement test

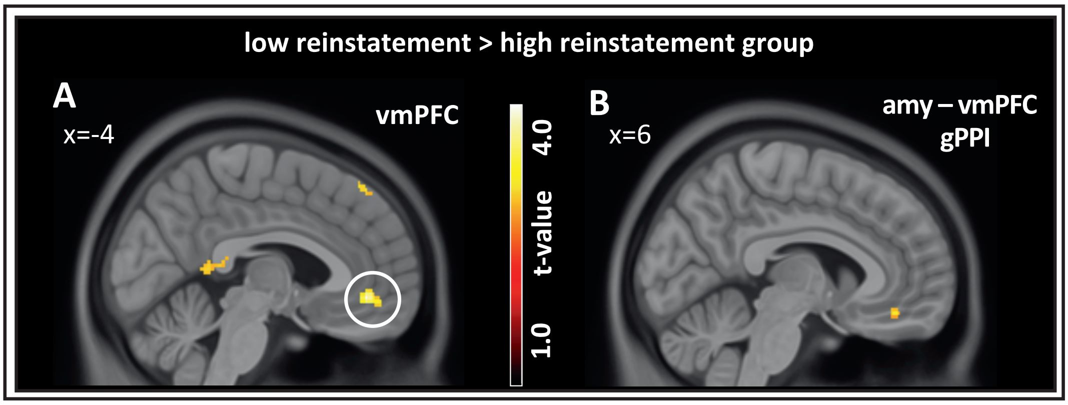

Figure S1. Subgroup analyses based on median split on differential SCRs during reinstatement test. A Higher differential BOLD responses (CS+ > CS-) in vmPFC in participants experiencing low opposed to high psychophysiological reinstatement ([x:-4, y:42, z:-8], Z=3.83, p_{FWE_ROI}=.011). **B** Cue-dependent functional amygdala–vmPFC connectivity (gPPI) during late reinstatement test was trendwise enhanced in the low compared to the high reinstatement group ([x:6, y:40, z:-16], Z=3.08, p_{FWE_ROI}=.077).

Supplementary Table S1: Exploratory whole-brain results during reinstatement test, displayed at p<.001 uncorrected using a cluster-forming threshold of k=10 contiguous voxels

**Contrast**	**Region**	**Side**	**Voxel**	**Peak voxel MNI**			$\mathbf{Z_{max}}$	$\mathbf{P_{uc}}$
				x	y	z		
Full reinstatement test [all CS+ > CS-]	Amygdala	L	22	-26	2	-26	3.82	<.001
	Cerebellum	L	14	-16	-38	-30	3.59	<.001
	Middle temporal gyrus	R	11	46	-62	16	3.55	<.001
	Precuneus	R	10	6	-58	22	3.49	<.001
Early reinstatement $_{[CS+ > CS-]}$ > late reinstatement $_{[CS+>CS-]}$	Middle frontal gyrus	R	23	22	6	42	4.21	<.001
	Putamen	R	16	18	8	-10	3.82	<.001
	Middle cingulum	L	14	-16	-34	34	3.71	<.001
	Middle temporal gyrus	L	15	46	-48	-6	3.70	<.001
	Supramarginal gyrus	R	16	64	-40	34	3.69	<.001
	Precuneus	R	29	10	-58	62	3.67	<.001
	Medial orbitofrontal gyrus	L	13	-8	60	-14	3.58	<.001
	Precentral gyrus	L	26	-50	4	26	3.44	<.001

Exploratory connectivity analysis using the right NAcc as seed region

Following a reviewer's suggestion, we further explored functional cue-dependent connectivity between right NAcc, which showed a time-dependent effect during the reinstatement test with stronger BOLD response towards CS+ compared to CS- during the early compared to the late reinstatement phase, and the vmPFC by applying a similar gPPI analysis as for the amygdala but using the right NAcc as seed region. This analysis revealed no significant cue-dependent NAcc-vmPFC involvement across or within phases of the reinstatement test ($p_{FWE\ ROI}$≥.648). Interestingly, we instead observed heightened functional connectivity between right NAcc and amygdala ([x:23, y:0, z:-26]; Z=3.40; $p_{FWE\ ROI}$=.035) during the early reinstatement phase, suggesting these two structures to closely interact upon CS+ compared to CS- presentation.

Effects of contingency awareness on conditioning measures

Following a worthwhile reviewer comment, we explored possible associations between contingency awareness and measured indices of conditioning on day 1. As our study was not designed to unambiguously classify participants as contingency aware or unaware, we based our analyses on a median split on difference scores of rated reward probabilities (CS+ *minus* CS-) acquired after conditioning (see Figure S2A). We then re-evaluated each conditioning measure by introducing a between subject group factor (aware vs. unaware), i.e. CS pleasantness ratings, SCRs, RTs, and HR were analyzed in separate mixed ANOVAs with within subject factors cue type (CS+ vs. CS-) and time (pre/early vs. post/late) and between subject factor group (aware vs. unaware), and startle responses were analyzed in a mixed

ANOVA with within subject factor cue type and between subject factor group. Significant interactions with contingency awareness were followed up by groupwise post-hoc analyses.

CS pleasantness ratings. Including awareness as an additional predictor revealed a significant cue x time interaction ($F(1,60)=4.60$, $p=.036$, $\eta^2 p=.07$), as well as a significant three-way interaction cue x time x group ($F(1,60)=4.97$, $p=.030$, $\eta^2 p=.08$; Figure S2B). No further main or interactions effects were observed ($F(1,60)\leq 2.48$, $p\geq .120$). Post-hoc ANOVAs showed a significant cue x time interaction only in aware subjects ($F(1,30)=5.62$, $p=.024$, $\eta^2 p$ $=.16$), while no significant main or interaction effects were present in the unaware group ($F(1,30)$ ≤ 0.72, $p\geq .402$), indicating that CS pleasantness ratings were mediated by contingency awareness.

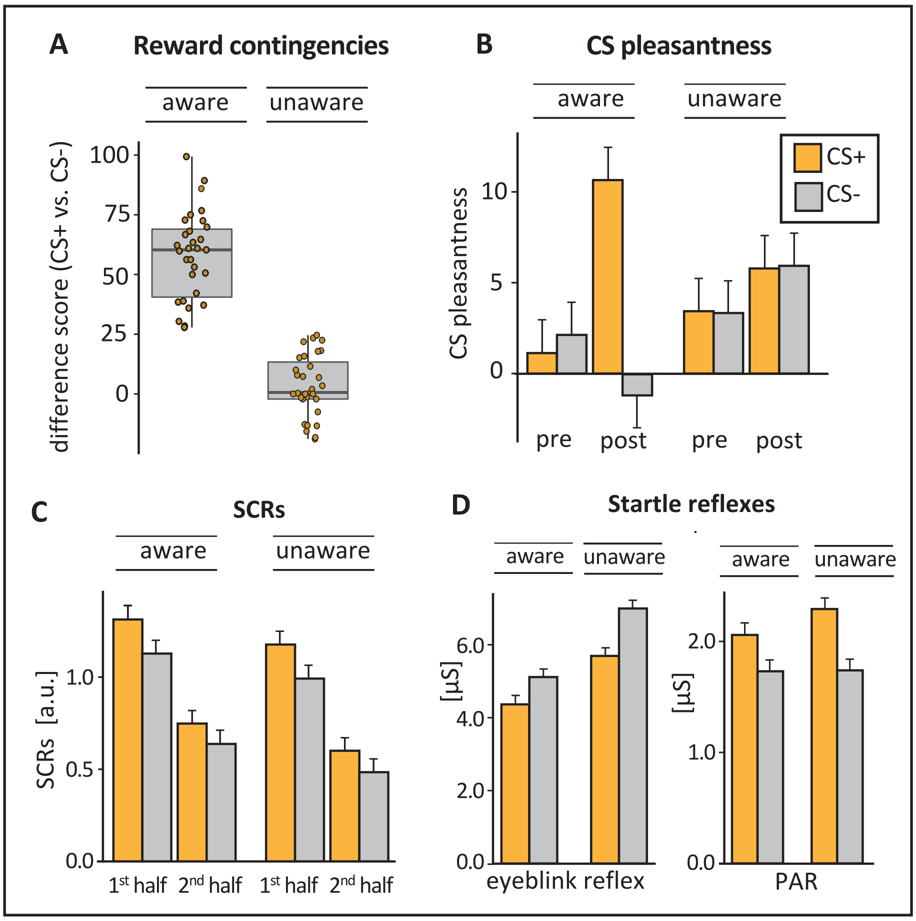

Figure S2. Effects of contingency awareness on conditioning measures on day 1. A. Classification of participants into contingency aware vs. unaware participants was based on a median split on difference scores of rated reward probabilities for each cue (CS+ *minus* CS-) obtained after conditioning. **B-D.** CS pleasantness, SCRs, and startle responses for contingency aware and unaware participants. Error bars represent within-subject SEM[3,4]; a.u., arbitrary units; PAR, postauricular reflex.

SCRs. Validating our main analysis, we observed a significant main effect of cue ($F(1,58)=6.96$, $p=.011$, $\eta^2 p=.11$) due to increased SCRs towards the CS+ compared to the

CS- and a main effect of time ($F(1,58)=21.78$, $p<.001$, $\eta^2p=.27$) due to overall decreasing SCRs over the conditioning session (Figure S2C). No further main or interaction effects were significant ($F(1,58)\leq0.63$, $p\geq.430$), suggesting that SCRs were unaffected by contingency awareness.

Startle responses. A significant main effect of cue confirmed differential modulation of both startle measures after conditioning (eyeblink reflex: $F(1,44)=10.13$, $p=.003$, $\eta^2p=.19$; PAR: $F(1,46)$, $p=.005$, $\eta^2p=.16$), while no main or interaction effects with awareness were observed for both measures (eyeblink reflex: $F(1,44)\leq1.32$, $p\geq.256$; PAR: $F(1,46)\leq0.56$, $p\geq.457$; Figure S2D).

RTs. In line with our main analysis, no significant main or interaction effects were observed in RTs towards cues (all $F(1,59)\leq3.93$, $p\geq.052$).

HR. Analysis of HR revealed only a main effect of time due to general HR increases over phases ($F(1,57)=8.08$, $p=.006$, $\eta^2p=.12$), but no significant conditioning effects or interactions with contingency awareness (all $F(1,57)\leq3.37$, $p\geq.072$).

References

1 Pan J, Tompkins WJ. A real-time QRS detection algorithm. *IEEE Trans Biomed Eng* 1985; **BME-32**: 230–236.

2 Sherozia OP, Ermishkin W, Lukoshkova E V. Dynamics of swallowing-induced cardiac chronotropic responses in healthy subjects. *Bull Exp Biol Med* 2003; **135**: 322–326.

3 Masson ME, Loftus GR. Using confidence intervals for graphically based data interpretation. *Can J Exp Psychol* 2003; **57**: 203–220.

4 Loftus GR, Masson ME. Using confidence intervals in within-participant designs. *Psychon Bull Rev* 1994; **1**: 476–490.
